# Mandibular Bony Exostoses or Hyperostosis: A Case Report

**DOI:** 10.7759/cureus.72941

**Published:** 2024-11-03

**Authors:** Akshaya Ravi, Ramachandra Reddy Gowda Venkatesha, Karthik Rajaram Mohan

**Affiliations:** 1 Oral Medicine and Radiology, Vinayaka Mission's Sankarachariyar Dental College, Vinayaka Mission's Research Foundation (DU), Salem, IND

**Keywords:** : alveolar bone, edentulous mandible, exostoses, osseous crater, palatal

## Abstract

Mandibular bony exostoses or hyperostoses are benign, non-neoplastic, localized bony outgrowths that are broad-based, slow-growing, and surface masses on the buccal or lingual surface of the mandible alveolar bone. Such exostoses grow over several years. Exostoses are more common in the maxilla posterior region along the maxillary tuberosity, called palatal exostoses. Torus palatinus occurs in the mid-line of the hard palate; mandibular torus or torus mandibularis occurs along the mandibular canine, premolar regions. We report a rare case of such bony exostoses on the lingual surface of the mandible in a 70-year-old edentulous male.

## Introduction

Mandibular bony exostoses are non-neoplastic, benign, painless, surface bony protuberant masses that increase patient concern about esthetics and compromise periodontal health by causing food lodgment [1]. Etiology still needs to be well established [2]. This bony lump or mass is attributed to an abnormal increase in masticatory forces on the teeth. They slowly increase in size in patients, increasing with age and time. Nery et al. stated that 276 of 681 dry skulls had palatal exostoses. The European, Asian, Mexican-Peruvian, and African dried skull samples had 45.54%, 47.25%, 27.27%, and 25%, respectively [3]. The prevalence rate of mandibular bony exostoses is 14.4% for oral exostoses and is observed in patients with bruxism and attrition [3]. Of 618 patients (264 women and 354 men) aged 10-82 years at the University of Jordan Hospital, Amman, Jordan, only 2.4% had palatal exostoses. The occurrence of exostoses occurs with increasing age [4]. Auškalnis et al. reported a prevalence of 1.8% among 162 twins (81 twin pairs) [5].

## Case presentation

**Figure 1:**
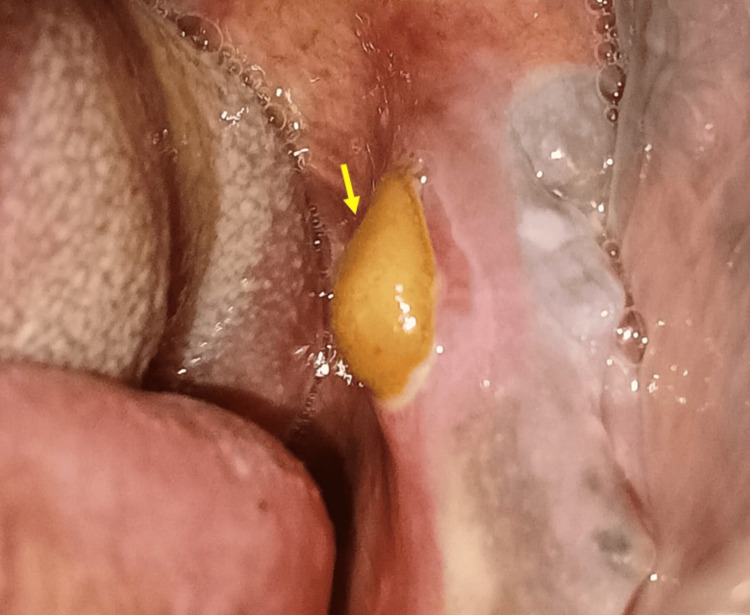
Intraoral clinical examination revealed a bony protuberance on the lingual aspect of the mandible (yellow arrow).

A 70-year-old male presented to our department for a routine dental check-up. His past medical history revealed no comorbidities such as uncontrolled diabetes or hypertension. His family history was non-contributory, with no harmful oral habits like chewing or smoking tobacco and no alcohol consumption. The past surgical history revealed that he underwent an uneventful dental extraction of his left mandibular posterior teeth three months ago. The past dental history revealed he had been a denture wearer for the past month but did not wear his denture regularly. The extraoral examination did not reveal similar masses on any part of his body. The intraoral soft tissue clinical examination revealed a whitish area on the edentulous alveolar ridge. On inspection, it was non-scrapable, non-tender, and suggestive of ridge keratosis due to a removable prosthesis. The hard tissue examination on inspection revealed a brownish-yellow-colored bony protuberance measuring about 1 x 1.5 cm on the lingual or palatal aspect of the posterior left mandibular alveolar bone concerning the mandibular second molar region above the mylohyoid ridge (Figure 1).

**Figure 2:**
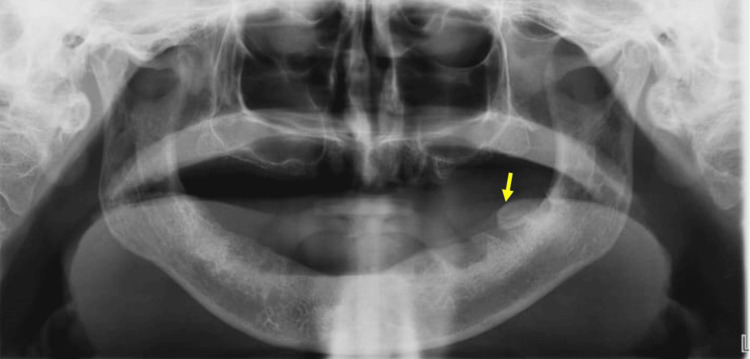
Orthopantomography revealed a radiopaque protuberance in the edentulous mandibular second molar region (yellow arrow).

The mass was bony-hard on palpation in consistency, sessile, and non-tender. Orthopantomography revealed a radiopaque mass in relation to the left mandibular second molar region. The contrast or density of the radiopaque mass blends with the adjacent cortical mandibular alveolar bone (Figure 2).

Based on the above clinical findings, a provisional diagnosis of bony exostoses was made. The differential diagnosis includes bone spurs, osteoma, osteosarcoma, and osteoradionecrosis. The osteonecrosis and osteoradionecrosis were ruled out based on the absence of a history of bisphosphonates or radiotherapy in our patient. The differential diagnosis of bony exostoses is enumerated (Table 1).

**Table 1:** Differential diagnosis of bony exostoses.

Clinical differential diagnosis	Clinical features
Hyperostoses or bony exostoses	Bony exostoses are a benign proliferation of cancellous bone. They occur in either the buccal or palatal aspect of the mandible. The density of such exostoses usually blends with the adjacent bone [1-4]
Dense Bone Island	An intraosseous counterpart of idiopathic osteosclerosis. They occur from internal bony growths of cancellous bone inside the mandibular cortical bone [5]
Bone spur	A localized area of hyperactivity in bone. They occur as firm, asymptomatic bony masses along the line of insertion of muscle fibers along the course of their action [6]
Chronic osteomyelitis	Low dense bone. A piece of necrotic bone (sequestrum) is usually detached from the bone. Proliferating reaction with elevation of the periosteum results in an onion-peel-like appearance on the outer surface of the cortical bone. History of traumatized or difficult dental extraction. Swelling with extraoral sinus, tenderness on palpation, and pathological fracture occur if left untreated [7]
Osteoma/osteochondroma	Usually attached to the outer cortical plate. They have a dense outer cortical border [8,9]
Osteosarcoma	Sunburst appearance is seen due to the rapid peeling of the outer periosteal covering of the affected bone. Poor prognosis [10]
Osteonecrosis	History of bisphosphonate therapy for osteoporosis [11].
Osteoradionecrosis	History of radiotherapy for the treatment of oral cancer [12].

Limitations

Conventional radiographs such as mandibular occlusal radiographs and specialized radiographic techniques such as multislice computed tomography (CT) and cone beam CT (CBCT) were not performed on this patient. In this case, the final diagnosis was based only on history and clinical findings. The patient did not consent to biopsy or surgical excision, as he was completely asymptomatic.

## Discussion

Exostoses are more common as a person grows older [13]. During the 10th to 13th week of gestation, endochondral ossification occurs around the protruded medial lamina of the mandible reaching Meckel's cartilage immediately before mylohyoid muscle attachment, resulting in bony prominence [13].

Exostosis development is a complicated process that can be triggered by any factor that causes gingival tissue injury and inflammation [14,15]. In genetically predisposed people, however, significant occlusal stress, which is frequently seen in TMJ dysfunction, is the key environmental component contributing to exostosis development [16]. Exostoses are bony overgrowths that can occur due to unusually high masticatory stresses on the teeth. They can grow to around 2-3 cm in diameter. They contribute to food lodgment, impairing periodontium [17]. Exostoses can occur as unilateral, painless masses, causing facial asymmetry.

Exostoses are reported along the mandibular angle. The authors pointed out the rarity of buccal exostosis on the mandibular angle as an anatomic location. Exostoses may form as a result of functional factors. In certain older adults, there is a growing association between severe exostoses and considerable tooth attrition.

According to the theory of subpontic hyperostosis, excessive occlusal stress causes the crystal alveolar bone to form beneath the pontic region, opposing the occlusion pressures of mastication [17]. Exostoses are lumps of calcified bones that result in facial deformity and are surgically removed following an intraoral approach [18]. Exostoses can be surgically removed with a mallet and chisel, and such exostoses are used as autografts to treat shallow bone crater defects [18]. Exostoses can occur in the condyle, resulting in clicking and pain in the temporomandibular joint [18]. Siegel et al. reported cases of exostoses following a skin vestibuloplasty procedure [19,20].

Clinical significance

The clinical significance of this case report is the presence of an exposed bone mimicking the clinical features of osteonecrosis or osteoradionecrosis and providing a diagnostic challenge to oral physicians. Hence, careful history-taking and clinical examination are essential to avoid unwanted fear and apprehension in the patient.

## Conclusions

A comprehensive, detailed history-taking and knowledge of clinical and radiographic features of exostoses are essential to rule out other differential diagnoses such as torus, bone spur, chronic osteomyelitis, osteonecrosis, and osteosarcomas that may warrant unnecessary biopsy or surgical procedures and alleviate unwanted apprehension and anxiety in the patient. No treatment is indicated for such exostoses unless it interferes with a function such as denture insertion or periodontal regeneration procedures requiring gingivectomy.
